# Current status of remote radiotherapy treatment planning in Japan: findings from a national survey^†^

**DOI:** 10.1093/jrr/rrad085

**Published:** 2023-11-22

**Authors:** Masahide Saito, Tetsuro Tamamoto, Shohei Kawashiro, Rei Umezawa, Masaki Matsuda, Naoki Tohyama, Yoshiyuki Katsuta, Takayuki Kanai, Hikaru Nemoto, Hiroshi Onishi

**Affiliations:** Department of Radiology, University of Yamanashi, 1110 Shimokato, Chuo, Yamanashi 409-3898, Japan; Department of Medical Informatics, Nara Medical University Hospital, 840 Shijyo-cho, Kashihara, Nara 634-8521, Japan; Department of Radiation Oncology, Faculty of Medicine, Yamagata University, 2-2-2 Iida-Nishi, Yamagata, Yamagata 990-9585, Japan; Department of Radiation Oncology, Tohoku University Graduate School of Medicine, 1-1 Seiryo-machi, Aoba-ku, Sendai, Miyagi 980-8574, Japan; Department of Radiology, University of Yamanashi, 1110 Shimokato, Chuo, Yamanashi 409-3898, Japan; Division of Medical Physics, Tokyo Bay Makuhari Clinic for Advanced Imaging, Cancer Screening, and High-Precision Radiotherapy, 1-17 Toyosuna, Mihama-ku, Chiba, Chiba 261-0024, Japan; Department of Radiation Oncology, Tohoku University Graduate School of Medicine, 1-1 Seiryo-machi, Aoba-ku, Sendai, Miyagi 980-8574, Japan; Department of Radiation Oncology, Tokyo Women's Medical University, 8-1, Kawada-Cho, Shinjuku-Ku, Tokyo 162-8666, Japan; Department of Radiology, University of Yamanashi, 1110 Shimokato, Chuo, Yamanashi 409-3898, Japan; Department of Radiology, University of Yamanashi, 1110 Shimokato, Chuo, Yamanashi 409-3898, Japan

**Keywords:** remote radiotherapy treatment planning, telework, survey, network, workforce

## Abstract

The purpose of this study was to investigate the status of remote-radiotherapy treatment planning (RRTP) in Japan through a nationwide questionnaire survey. The survey was conducted between 29 June and 4 August 2022, at 834 facilities in Japan that were equipped with linear accelerators. The survey utilized a Google form that comprised 96 questions on facility information, information about the respondent, utilization of RRTP between facilities, usage for telework and the inclination to implement RRTPs in the respondent’s facility. The survey analyzed the utilization of the RRTP system in four distinct implementation types: (i) utilization as a supportive facility, (ii) utilization as a treatment facility, (iii) utilization as a teleworker outside of the facility and (iv) utilization as a teleworker within the facility. The survey response rate was 58.4% (487 facilities responded). Among the facilities that responded, 10% (51 facilities) were implementing RRTP. 13 served as supportive facilities, 23 as treatment facilities, 17 as teleworkers outside of the facility and 5 as teleworkers within the facility. In terms of system usage between supportive and treatment facilities, 70–80% of the participants utilized the system for emergencies or as overtime work for external workers. A substantial number of facilities (38.8%) reported that they were unfamiliar with RRTP implementation. The survey showed that RRTP utilization in Japan is still limited, with a significant number of facilities unfamiliar with the technology. The study highlights the need for greater understanding and education about RRTP and financial funds of economical compensation.

## INTRODUCTION

Viewed from a macroscopic perspective, the unequal spatial distribution of human resources and equipment poses a significant challenge in the endeavor to harmonize the quality of radiotherapy. On the other hand, from a microscopic perspective that focuses on each facility, the enhancement of the work environment and operational efficiency of each staff member assumes critical importance, as does addressing the shortage of temporary personnel stemming from factors such as vacation time, to provide efficient and safe radiotherapy. To tackle these issues, remote-radiotherapy treatment planning (RRTP) is deemed a valuable tool [1]. To this end, the proper execution of real-time RRTP necessitates remote operations, such as target volume delineation and treatment planning, that are conducted under the careful supervision and guidance of more experienced colleagues at the supporting facility [1]. For example, in Japan, although RRTP for emergency is allowed in the national health insurance system, at least one full-time radiation oncologist (RO) with at least 5 years of experience is an institutional requirement for the RRTP [2].

In the 2000s, the RRTP system in Norway had reportedly imposed a communication speed restriction of 2 Mbps [3]. However, presently, the communication speed has undergone a remarkable advancement, clocking at roughly 500 Mbps via an internet service provider. Furthermore, the use of the system has been extended to a variety of applications. Especially, web-based quality assurance (QA) systems for radiotherapy have been extensively studied [4, 5], and in recent years, novel remote approaches, such as cloud-based peer review systems, have been adopted by healthcare facilities and regions [6].

Moreover, due to the recent spread of Covid-19 virus, there have been scattered overseas reports that propose RRTP during a pandemic. The International Atomic Energy Agency has presented telemedicine interventions for radiation therapy processes [7], which include the process for volume delineation, treatment planning and setup verification. Moreover, it has been proposed that clinical physicists could entirely carry out remote plan reviews and QA processes, especially during pandemic situations, for the medical physics department [8, 9]. In fact, there have been several reports of the effective utilization of remote technology in the USA, China, Denmark and Iran during the Covid-19 pandemic [9–13]. In Japan, it has been reported that the Covid-19 pandemic led to various changes in radiotherapy, such as a decrease in the number of patients and an increase in the use of hypofractionated radiotherapy [14]. RRTP was also considered effective under these circumstances; however, the status of its adoption remained uncertain and called for further investigation.

In Japan, RRTP was first implemented in the early 2000s [15]. Furthermore, since 2018, the utilization of RRTP for emergency cases has been covered by insurance [2]. However, a comprehensive survey of the actual utilization of RRTP across Japan has not yet been conducted. On the contrary, no such survey on the RRTP has been conducted worldwide to date. Therefore, the purpose of this study was to investigate the status of RRTP in Japan through a nationwide questionnaire survey.

## MATERIALS AND METHODS

### Definition of RRTP

We classified the utilization of RRTP into four distinct implementation types: (i) utilization as a supportive facility, (ii) utilization as a treatment facility, (iii) utilization as a teleworker outside of the facility and (iv) utilization as a teleworker within the facility. Each implementation type was scrutinized. First, utilization as a supportive facility (Type 1) pertains to its use as a means of supporting radiotherapy at a partner hospital in a facility such as a regional flagship hospital that possesses a comparatively complete staff. Utilization as a treatment facility (Type 2) refers to cases where the facility is employed for the purpose of receiving support from a partner core hospital in a facility that provides radiotherapy, wherein there is a scarcity of ROs and similar personnel. The prevailing assumption is that such hospitals typically lack a full-time RO on their staff. Utilization as a teleworker outside of the facility (Type 3) refers to cases in which RRTP is personally performed outside the facility (e.g. at home or others). Finally, utilization as an in-hospital teleworker (Type 4) describes cases where RRTP is conducted in a separate room from the treatment planning room within the facility.

### Conducting the survey

The survey was carried out between 29 June and 4 August 2022, at 834 facilities in Japan that were equipped with linear accelerators. For the scope of the survey, a team of researchers curated each technical question with a focus on the utilization of RRTP across facilities (i.e. supportive and treatment facilities) and individual practices (such as telework). Respondents were asked to answer only in relation to the technology they use. The survey also extended to all facilities, even those not currently implementing RRTP, to gather information about their human resources, interest in future adoption of RRTP and the obstacles to its implementation. To maximize the response rate, the survey was designed to allow a representative from each facility to participate, including not just ROs but anyone (medical physicist [MP], radiotherapy technologist [RTT], etc.) involved in radiotherapy. A Google form was utilized to administer the questionnaire, which encompassed a total of 96 questions. The specifics of the questionnaire items are outlined in Supplemental Document 1.

## RESULTS

The survey response rate yielded 58.4% (487 facilities responded). Table 1 demonstrates the survey outcome across all facilities. Among the facilities that responded, 10% (51 facilities) were implementing RRTP, with 13 serving as supportive facilities, 23 as treatment facilities, 17 as teleworkers outside of the facility and 5 as teleworkers within the facility. Regarding the usefulness of RRTP, 65.5% of the facilities, including those responding Yes and Moderately Yes, considered it a valuable tool to enhance the number of high-precision radiotherapy patients. Moreover, 37% of the facilities, comprising those that have already implemented RRTP, expressed an interest in utilizing it in the future, while 24.2% of the facilities did not require it. Conversely, a substantial number of facilities (38.8%) reported that they were unfamiliar with RRTP implementation.

**Table 1:** Survey results for all facilities

Contents	*n* (%)
Survey facilities	834 (100%)
Responding facilities	487 (58%)
Occupation (*n* = 487)	
Radiation oncologist	306 (63%)
Medical physicist	33 (7%)
Radiotherapy technologist	146 (30%)
Other	2 (0%)
Whether RRTP has already been implemented (Yes) (*n* = 487)	51 (10%)
(a) Utilization as a supportive facility (duplicate possible)	13 (3%)
(b) Utilization as a treatment facility (duplicate possible)	23 (5%)
(c) Utilization as a teleworker outside of the facility (duplicate possible)	17 (3%)
(d) Utilization as a teleworker within the facility (duplicate possible)	5 (1%)
Do you think remote technology can be a useful tool to increase the number of high precision radiotherapy patients at your institution? (*n* = 487)	
Yes	124 (25.5%)
Moderate yes	195 (40.0%)
Moderate no	105 (21.6%)
No	63 (12.9%)
Are you considering implementing RRTP in the future? (*n* = 487)	
Already implemented or planning to implement	58 (11.9%)
No plans, but would like to	122 (25.1%)
Not necessary	118 (24.2%)
Unfamiliar	189 (38.8%)

RRTP = remote-radiotherapy treatment planning.

Table 2 presents the survey results pertaining to supportive and treatment facilities. Regarding system usage, 70–80% of the participants utilized the system for emergencies or as overtime work for external workers. The most frequently employed procedures were contouring, beam setting and dose calculation. However, <50% of the facilities had adopted optimization, and the utilization of QA was also low.

**Table 2:** Survey result for supportive facilities and treatment facilities

	Supportive facility (*n* = 13)	Treatment facility (*n* = 23)
Usage (duplicate possible)		
Emergencies	11 (85%)	18 (78%)
As overtime work for external workers	11 (85%)	16 (70%)
Confirmation of need for replanning	6 (46%)	7 (30%)
Confirmation of treatment plans for nonspecialist doctors	7 (54%)	3 (13%)
QA/QC	2 (15%)	1 (4%)
Process (duplicate possible)		
Evaluation and confirmation of dose distribution	12 (92%)	20 (87%)
Contouring (normal organs)	10 (77%)	19 (83%)
Contouring (target volume)	11 (85%)	18 (78%)
Beam setting (3DCRT or IMRT)	11 (85%)	20 (87%)
Dose calculation (3DCRT or IMRT)	11 (85%)	20 (87%)
Optimization (IMRT)	2 (15%)	10 (43%)
QA/QC	2 (15%)	1 (4%)
Research	0 (0%)	1 (4%)
Questions about supportive facility		
Number of connected treatment facility	1 facility: 5 (38%) 2 facility: 5 (38%) 3 facility: 1 (8%) 4 facility: 1 (8%) 5 facility: 1 (8%)	
Reward for RRTP	No compensation: 7 (54%) Hourly compensation for planners: 3 (23%) Remuneration per number of cases for planners: 2 (15%) Compensation per number of cases for support facilities: 1 (8%)	
Questions about treatment facility		
Percentage of remote-treatment planning used for all patients		5%>:11 (48%) 5–25%: 7 (30%) 26–50%: 2 (9%) 51–75%: 2 (9%) 75%<: 1 (4%)
The output dose of linear accelerators was evaluated by a third party (Yes)		17 (74%)

IMRT = intensity modulated radiotherapy, 3DCRT, 3D conformal radiotherapy, QA = quality assurance, QC = quality control, RRTP=remote-radiotherapy treatment planning.

In relation to supportive facilities, 38% of them were linked to a solitary treatment facility, while the others were linked to multiple treatment facilities. More than half of the facilities did not receive reward for implementing RRTP.

As for treatment facilities, roughly half of them employed RRTP for <5% of all patients, indicating limited use of this modality. Moreover, third-party output evaluation organizations conducted output dose surveys [16, 17] at 74% of the facilities to ensure the quality of radiotherapy. This is an essential requirement for safe irradiation at treatment facilities using RRTP.

Table 3 shows the results for teleworkers outside of the facility and within the facility. The primary application of the system was to enhance the efficiency of ROs (41 and 80%, respectively). Subsequently, there were enhancements observed in the work of MPs and Covid-19 mitigation measures. The techniques employed varied from contouring, beam setting, dose calculation to optimization calculation for teleworkers outside the facility. Conversely, for teleworkers within the facility, it appeared to be primarily employed for contouring of normal organs and optimization calculations.

**Table 3:** Survey result for teleworkers outside of the facility and within the facility

	Teleworker outside of the facility (*n* = 17)	Teleworker within the facility (*n* = 5)
Usage (duplicate possible)		
To enhance the efficiency of ROs	7 (41%)	4 (80%)
To enhance the efficiency of MPs	4 (24%)	2 (40%)
Address Covid-19 pandemic	3 (18%)	1 (20%)
To support shorter working hours	3 (18%)	0 (0%)
To provide work opportunities during maternity/paternity leave	1 (6%)	0 (0%)
Others	4 (24%)	0 (0%)
Process (duplicate possible)		
Evaluation and confirmation of dose distribution	14 (82%)	4 (80%)
Contouring (normal organs)	11 (65%)	5 (100%)
Contouring (target volume)	12 (71%)	3 (60%)
Beam setting (3DCRT or IMRT)	12 (71%)	1 (20%)
Dose calculation (3DCRT or IMRT)	12 (71%)	1 (20%)
Optimization (IMRT)	9 (53%)	5 (100%)
QA/QC	5 (29%)	1 (20%)
Research	0 (0%)	1 (20%)

IMRT = intensity modulated radiotherapy, 3DCRT = 3D conformal radiotherapy, QA = quality assurance, QC = quality control, RRTP=remote-radiotherapy treatment planning.

Supportive facilities are required to prepare client treatment planning systems that are connected to the treatment facility. Moreover, the system must be linked from the treatment planning room to individual workrooms via a network during telework. Table 4 presents the treatment planning system utilized for RRTP, as executed by 51 facilities. Eleven different treatment planning systems (Pinnacle, Monaco, RayStation, Eclipse, Precision, Planning Station, MultiPlan, iPlan, Elements, XiO and Oncentra) were used for various purposes. Note that, as of writing, all treatment planning systems investigated in this study were approved medical devices. However, the remote use of these devices was left to the discretion of the facilities, and the ultimate responsibility rested with each facility rather than with the manufacturer.

**Table 4:** Survey result for treatment planning system for remote planning

	Treatment planning system for remote planning (duplicate possible)			
	Supportive facility (*n* = 13)	Treatment facility (*n* = 23)	Teleworker outside of the facility (*n* = 17)	Teleworker within the facility (*n* = 5)
Pinnacle	5 (38%)	6 (26%)	2 (12%)	0 (0%)
Monaco	4 (31%)	6 (26%)	1 (6%)	3 (60%)
RayStation	3 (23%)	5 (22%)	3 (18%)	1 (20%)
Eclipse	2 (15%)	3 (13%)	11 (65%)	1 (20%)
Precision	2 (15%)	0 (0%)	1 (6%)	0 (0%)
Planning Station	0 (0%)	2 (9%)	0 (0%)	1 (20%)
MultiPlan	1 (8%)	1 (4%)	1 (6%)	1 (20%)
iPlan	0 (0%)	0 (0%)	1 (6%)	0 (0%)
Elements	0 (0%)	0 (0%)	1 (6%)	0 (0%)
XiO	1 (8%)	4 (17%)	0 (0%)	0 (0%)
Oncentra	0 (0%)	0 (0%)	1 (6%)	0 (0%)

Table 5 shows the survey results on cybersecurity, communication tools and attendance management among supportive and treatment facilities. Security guidelines had been developed in half of both supportive and treatment facilities. About 30% of the facilities indicated that they obtained patient information through remote access to hospital information systems. In >60% of the facilities, patient consent for the use of RRTPs had not been obtained. Telephone and e-mail were the communication tools used by >70% of the respondents. In the use of RRTP, the most common team composition involved a ROs paired with a RTT. This was followed by combinations of a RO with a nonspecialist doctor, and then by a RO with a MP. In addition, >80% of facilities did not implement time and attendance management. Furthermore, more than half of the facilities had no compensation.

**Table 5:** Survey results on cybersecurity, communication tools and attendance management among supportive and treatment facilities

Factors	Answers	Supportive facility (*n* = 13)	Treatment facility (*n* = 23)
Security guidelines for RRTP at the facility	Available	7 (54%)	10 (45%)
	Not available	6 (46%)	12 (55%)
Means of obtaining patient information for treatment facilities	Remote access to hospital information systems	4 (31%)	4 (18%)
	Providing the necessary information with anonymization	1 (8%)	3 (14%)
	Providing the necessary information without anonymization	2 (15%)	4 (18%)
	Not provided	2 (15%)	8 (36%)
	Others	4 (31%)	3 (14%)
Explanation to the patient about RRTP	Explained (with consent form)	2 (15%)	3 (13%)
	Explained (without written consent)	1 (8%)	2 (9%)
	Not explained	9 (69%)	15 (65%)
	Unknown	1 (8%)	3 (13%)
Communication tools between facilities (duplicate possible)	Telephone	10 (77%)	19 (83%)
	E-mail	9 (69%)	18 (78%)
	Web conference system	2 (15%)	8 (35%)
	Business chat tool	1 (8%)	0 (0%)
	Fax	1 (8%)	1 (4%)
	Not used	1 (8%)	0 (0%)
Combination to contact when using RRTP ‘Supportive facility - Treatment facility’ (duplicate possible)	RO–RTT	10 (77%)	10 (43%)
	RO–nonspecialist doctor	7 (54%)	2 (9%)
	RO–MP	4 (31%)	6 (26%)
	RO–RO	3 (23%)	4 (17%)
	Nonspecialist doctor–RTT	2 (15%)	0 (0%)
	Nonspecialist doctor–nonspecialist doctor	1 (8%)	0 (0%)
	Nonspecialist doctor–MP	1 (8%)	0 (0%)
	MP–RTT	1 (8%)	0 (0%)
	RTT–nonspecialist doctor	1 (8%)	0 (0%)
	RTT–RTT	1 (8%)	0 (0%)
	RTT–RO	0 (0%)	3 (13%)
	MP–MP	0 (0%)	2 (9%)
Attendance management	Regularly	2 (15%)	3(13%)
	Each time of use	4 (31%)	1(4%)
	Not conducted	7 (54%)	19 (83%)
Reward for RRTP	Compensation for the planner (per hour)	3 (23%)	2 (9%)
	Compensation for the planner (per case)	2 (15%)	1 (4%)
	Compensation for the support facility (per case)	1 (8%)	0 (0%)
	No compensation	7 (54%)	17 (74%)
	Unknown	0 (0%)	3 (13%)

RRTP = remote-radiotherapy treatment planning, RO = Radiation Oncologisit, RTT = Radiotherapy technologist, MP = Medical physicist.

Table 6 shows the survey results on cybersecurity, communication tools and attendance management for teleworkers. Security guidelines had been developed for half of the teleworkers outside the facility. For teleworkers, trends concerning the means of obtaining patient information, securing patient consent for the use of RRTPs and utilizing communication tools were similar to those outlined in Table 5. ROs accounted for >80% of the occupations using RRTP, while MPs accounted for >40%. In terms of where RRTPs were used, 82% were used at home and 53% at destination. Among teleworkers within the facility, 40% utilized RRTP in the examination room, while another 40% did so at their individual desks. In addition, >70% of teleworkers did not implement time and attendance management. Furthermore, >80% of the teleworkers had no compensation.

**Table 6:** Survey results on cybersecurity, communication tools and attendance management for teleworkers

Factors	Answers	Teleworker outside of the facility (*n* = 17)	Teleworker within the facility (*n* = 5)
Security guidelines for RRTP at the facility	Available	9 (53%)	0 (0%)
	Not available	8 (47%)	5 (100%)
Means of obtaining patient information for treatment facilities	Remote access to hospital information systems	6(35%)	
	Providing the necessary information with anonymization	3 (18%)	
	Providing the necessary information without anonymization	1 (6%)	
	Not provided	7 (41%)	
	Others	0 (0%)	
Explanation to the patient about RRTP	Explained (with consent form)	1 (6%)	
	Explained (without written consent)	2 (12%)	
	Not explained	13 (76%)	
	Unknown	1 (6%)	
Communication tools between facilities (duplicate possible)	Telephone	8 (41%)	
	E-mail	12 (71%)	
	Web conference system	4 (24%)	
	Business chat tool	0 (0%)	
	Fax	0 (0%)	
	Not used	4 (24%)	
Occupations that use RRTP (duplicate possible)	RO	14 (82%)	4 (80%)
	Nonspecialist doctor	3 (18%)	1 (20%)
	MP	7 (41%)	3 (60%)
	RTT	0 (0%)	2 (40%)
Locations that use RRTP (duplicate possible)	Home	14 (82%)	
	Examination room (in hospital)		2 (40%)
	individual desk (in hospital)		2 (40%)
	Others (out of hospital)	12 (71%)	
	Others (in hospital)		1 (20%)
Attendance management	Regularly	3 (18%)	
	Each time of use	1 (6%)	
	Not conducted	13 (76%)	
Reward for RRTP	Compensation for the planner (per hour)	1 (6%)	
	Compensation for the planner (per case)	2 (13%)	
	Compensation for the support facility (per case)	0 (0%)	
	No compensation	13 (81%)	
	Unknown	0 (0%)	

RRTP = remote-radiotherapy treatment planning, RO = Radiation Oncologisit, RTT = Radiotherapy technologist, MP = Medical physicist.

Table 7 shows the potential factors preventing the implementation of RRTP. Responses were given in the form of answers to preprepared choices. In descending order, the most common reasons were installation cost (73%), security aspect (67%), maintenance cost (62%), unclear responsibilities (41%), network communication speed (34%), medical information department decisions (19%), lack of support facilities (19%) and unable to ascertain need for support (11%).

**Table 7:** Potential factors preventing the implementation of RRTP

Factors	*n* = 487
Installation cost	354 (73%)
Security aspect	328 (67%)
Maintenance cost	301 (62%)
Unclear responsibility	200 (41%)
Network communication speed	164 (34%)
Medical information department decision	91 (19%)
Lack of support facilities	72 (15%)
Unable to ascertain need for support	56 (11%)

## DISCUSSION

Although there exist scattered references [18–20] demonstrating the utility of remote diagnosis for patients undergoing radiation therapy planning and case reviews via video conferencing [13, 21], there are few reports on the use of detailed remote radiotherapy treatment planning (RRTP) or surveys on their actual utilization. This investigation introduces the current situation in Japan by classifying its use into four categories of RRTP.

Initially, we focus on its application as a supportive and treatment facility. For supportive facilities, large hospitals such as cancer centers and university hospitals typically play this role. In Japan, ROs and MPs from such large hospitals occasionally go to work in small hospitals and clinics as treatment facilities to provide support. RRTP may be utilized when work that cannot be completed during an outpatient shift is brought back to the support facility to perform the task or when support is provided from within the support facility in an emergency or other situation. Additionally, the results exhibited that RRTP was employed for the assessment of the treatment plans created by inexperienced staff at treatment facilities. A survey of quality indicators for radiotherapy in Japan has revealed variations in the quality of treatment among different facilities [22]. The implementation of RRTP to connect these facilities could potentially facilitate the standardization of treatment quality on a nationwide scale. Another specific issue with the use of the facility as a support and treatment facility is contract status. In fact, as also highlighted in Table 7, compensation, hours of use and security systems should be carefully negotiated among facilities before use.

Next, we focus on its use as telework. The telework outside of the facility is generally used when a RO or MP is unable to be in the facility for some reason (e.g. on parental leave, business trip, COVID-19 measures, etc.) and cannot implement the treatment planning. From this survey, possible locations of use RRTP include home (82%) and others (71%). When used outside the facility, adequate consideration should be given to security aspects. The telework within the facility is possible by accessing the treatment planning equipment from outside the treatment planning room, which may be effective in improving work efficiency and preventing Covid-19 infection among coworkers.

In the field of medicine, the implementation of ‘tele’ technology has been pervasive across various domains [23], and its utilization has been expedited, particularly during the Covid-19 pandemic [24]. Specifically, the application of this technology has been well-established in the discipline of pathology [25]. Additionally, remote diagnostic imaging services have long been available for radiology [26] . Although radiotherapy cannot be wholly conducted in a remote manner due to the need for the physician to physically evaluate the patient and perform irradiation face-to-face, certain digitalized tasks such as treatment planning can be executed remotely. It has also been proposed that quality control services for medical physics can be remotely administered [27] . Furthermore, the current situation of the COVID-19 pandemic has generated numerous recommendations to reduce contact between patients and staff, including the promotion of hypofractionation, thereby making telemedicine and RRTP increasingly vital in the future [28–32]. Reportedly, 60% of facilities in the USA have implemented RRTP [33]. Conversely, the significance of on-site treatment planning has also been revalidated, underscoring the importance of striking a harmonious balance between the two modes of intervention [33].

Regarding treatment planning, it is feasible to execute all the steps ranging from contouring to optimization calculations externally, either interfacility or through telework. Some facilities have already demonstrated the successful utilization of this technology. Additionally, a significant number of facilities not currently implementing this system are receptive to its introduction. It is therefore imperative to generate additional evidence to support these facilities, even though we have mentioned the balance of online and on-site planning. However, a considerable proportion of facilities (~40%) responded with ‘unfamiliar’ when queried about the implementation of RRTP. This suggests that more opportunities to promote the benefits of this technology are necessary to facilitate its adoption. In particular, since security is a salient issue for telework, it is incumbent to examine prior successful implementations of this technology and develop comprehensive guidelines for their use [34, 35].

To the best of our knowledge, this is the first study to investigate various aspects of RRTP in Japan. Based on our findings, we propose the following recommendations for the future expansion of tele-radiotherapy:

- *To increase interfacility collaborations using remote techniques to support equalization and improvement for the quality of radiotherapy.*
- *To utilize remote techniques to enhance staffing efficiency and reform working systems in radiotherapy within a single facility.*
- *To develop the infrastructure for tele-radiotherapy both at the national and each institution levels.*

Furthermore, policies and research that take them into account are mandatory.

There were some limitations in this study. First, the limitation of this study is reliant on self-disclosure. Second, the difference of penetration rates of RRTP before and after the COVID-19 pandemic was not examined. Third, assuming the required sample size to be all mailing facilities (834 facilities) and a confidence level of 95% (*λ* = 1.96), the response rate for the present study was 58.4%, which gave an acceptable margin of error of 3.39%. Therefore, the data were considered to adequately reflect the national situation; however, as the responses were not obtained by random allocation, response bias was possible due to staff sufficiency at each facility, regional characteristics, etc. Fourth, although this study did investigate security and the acquisition of patient information, it did not explore specific types of individual connections such as VPNs and remote desktop connections, nor their robustness. Therefore, additional research is required in the future. Furthermore, since this research was limited to a survey pertaining to the status of RRTP, future investigations should focus on exploring the effective utilization models of RRTP, including those advanced by affiliated organizations in the field of radiotherapy. Our intention is to undertake further investigations on the effective application of RRTP in the future.

## CONCLUSION

This study elucidates the implementation of RRTP. It presents the first revelation of the factual adoption of RRTP in Japan. Roughly 10% of facilities in Japan employed the technology, and despite their recognition of its necessity and utility, its actual usage remained limited due to various challenges that needed to be addressed prior to its implementation and economic compensation.

## Supplementary Material

Supplemental_Document_1_rrad085

## Data Availability

The datasets generated and/or analyzed during the current study are available from the corresponding author on reasonable request.
