# Supplementary material for: Current status of remote radiotherapy treatment planning in Japan: findings from a national survey
Source: J Radiat Res. 2023 Nov 22;65(1):127–35. doi: 10.1093/jrr/rrad085 (PMC10803164; doi:10.1093/jrr/rrad085)
Supplement: Supplemental_Document_1_rrad085 [file supplemental_document_1_rrad085.docx]

**Survey on Remote Radiotherapy Planning**

The name of the facility will not be disclosed, and the results of the survey will be disseminated at conferences, papers, etc. We will ask for one response per facility.

**General information**

1. Name of a prefecture
2. Name of Facility
3. Name of person filling out the form
4. Occupation of the person filling out the form
5. Number of Full-Time Physicians in the Radiotherapy Department
6. Number of Part-time Physicians in the Radiotherapy Department
7. Number of full-time radiological technologist in the radiotherapy department
8. Number of full-time quality control personnel (e.g., medical physicist) in the radiotherapy department
9. Facility Classification
10. Radiotherapy planning providers at your hospital (Multiple selections allowed)
11. Please tell us about the status of human resources involved in radiotherapy planning at your hospital.
12. Notification of additional fee for Remote Radiotherapy Planning
13. Please indicate all of the ways in which Remote Radiotherapy Planning is being implemented.

**Questions for facilities that are implementing the program as supportive facilities**

1. Number of treatment facilities connected to Remote Radiotherapy Planning
2. Number of physicians using Remote Radiotherapy Planning (total number of physicians)
3. Number of people using Remote Radiotherapy Planning (Total number of people assisting in planning (medical physicists, radiology technicians, etc.))
4. Did you set up an In-hospital working groups consisting of multiple professions for the launch of remote connectivity?
5. Who was primarily responsible in the Radiotherapy Department for setting up the remote connectivity? (Multiple selections allowed)
6. Did you receive any assistance from the facility administration, medical information office, etc. other than the Radiation Therapy Department in making the connection?
7. If you answered "Yes" to the above question, please provide the name of the specific department. If you know the job title of the person in charge (e.g., person in charge of system information, person in charge of safety management of medical information systems, medical information technologist, etc.), please indicate it as well.
8. In making the connection, was there a written agreement (including memorandum of understanding, etc.) with the treatment facility regarding the operation of the business?
9. Responsibilities for work with Remote Radiotherapy Planning
10. Uses of Remote Radiotherapy Planning (Multiple selections allowed)
11. Who is responsible for the cost of building the system?
12. Who is responsible for network connection costs?
13. How are performance fees handled for Remote Radiotherapy Planning?
14. Which of the following treatment planning devices implements Remote Radiotherapy Planning?
15. Does your hospital have security guidelines for Remote Radiotherapy Planning?
16. How do you share patient information for treatment planning?
17. Does your hospital maintain records of usage (users, usage time, etc.)?
18. Please select the combination of professionals who communicate with your hospital and the treatment facility regarding the status of treatment planning (Multiple selections allowed) (left: your hospital - right: treatment facility).
19. Do you have any tools that you use to communicate with the treatment facility at the time of use or to communicate with the treatment facility? (Multiple selections allowed)
20. Is there any prior explanation to the patient about the implementation of remote radiotherapy?
21. Which process is implemented in remote treatment plan? (Multiple selections allowed)
22. Regarding the remote treatment plan developed at your institution, how is the treatment plan confirmed (margins, prescribed dose, dose constraints, etc.)? (Multiple selections allowed)
23. How do you confirm the results of dose verification conducted by the treatment facility for the remote treatment plan developed by your hospital?
24. How will physician confirmation of the remote treatment plan developed by your hospital be performed for IGRT (including linear angiography) performed by the treatment facility?
25. Do you think that the quality of treatment and planning devices in treatment facilities should be regularly checked on-site by your hospital's quality control staff?

**Questions for facilities that are implementing the program as a treatment facility**

1. Number of support facilities to which Remote Radiotherapy Planning is connected.
2. Did you receive any assistance from the facility administration, medical information office, etc. other than the Radiation Therapy Department in making the connection?
3. If you answered "Yes" to the above question, please provide the name of the specific department. If you know the job title of the person in charge (e.g., person in charge of system information, person in charge of safety management of medical information systems, medical information technologist, etc.), please indicate it as well.
4. In making the connection, was there a written agreement (including memorandum of understanding, etc.) with the treatment facility regarding the operation of the business?
5. Responsibilities for work with Remote Radiotherapy Planning
6. Uses of Remote Radiotherapy Planning (Multiple selections allowed)
7. Who is responsible for the cost of building the system?
8. Who is responsible for network connection costs?
9. How are performance fees handled for Remote Radiotherapy Planning?
10. Approximately what percentage of all Remote Radiotherapy Planning is done remotely? (This includes cases where only part of the planning process is used.)
11. Which of the following treatment planning devices implements Remote Radiotherapy Planning?
12. Does your hospital have security guidelines for Remote Radiotherapy Planning?
13. How do you share patient information for treatment planning?
14. Does your hospital maintain records of usage (users, usage time, etc.)?
15. Please select the combination of professionals who communicate with your hospital and the treatment facility regarding the status of treatment planning (Multiple selections allowed) (left: your hospital - right: treatment facility).
16. Do you have any tools that you use to communicate with the treatment facility at the time of use or to communicate with the treatment facility? (Multiple selections allowed)
17. Is there any prior explanation to the patient about the implementation of remote radiotherapy?
18. Which process is implemented in remote treatment plan? (Multiple selections allowed)
19. Regarding the remote treatment plan developed at your institution, how is the treatment plan confirmed (margins, prescribed dose, dose constraints, etc.)? (Multiple selections allowed)
20. How do you confirm the results of dose verification conducted by the treatment facility for the remote treatment plan developed by your hospital?
21. How will physician confirmation of the remote treatment plan developed by your hospital be performed for IGRT (including linear angiography) performed by the treatment facility?
22. Do you think that the quality of treatment and planning devices in treatment facilities should be regularly checked on-site by your hospital's quality control staff?
23. Availability of output dose survey by a third-party output evaluation organization
24. Questions for facilities with individuals who are implementing the program as individuals (in-hospital teleworkers)
25. Did you receive any assistance from the facility administration, medical information office, etc. other than the Radiation Therapy Department in making the connection?
26. If you answered "Yes" to the above question, please provide the name of the specific department. If you know the job title of the person in charge (e.g., person in charge of system information, person in charge of safety management of medical information systems, medical information technologist, etc.), please indicate it as well.
27. Who are the occupations that use Remote Radiotherapy Planning as an individual (in-hospital teleworker)? (multiple selections allowed)
28. Uses of Remote Radiotherapy Planning (Multiple selections allowed)
29. Who is responsible for the cost of building the system?
30. Which of the following treatment planning devices implements Remote Radiotherapy Planning?
31. Does your hospital have security guidelines for Remote Radiotherapy Planning (individual ( in-hospital telework))?
32. Which process is implemented in remote treatment plan? (Multiple selections allowed)
33. Where is the remote treatment plan to be implemented? (All options must be independent of the main treatment planning office) (Multiple selections allowed)

**Questions for facilities with individuals (at home or out of home)**

1. Did you receive any assistance from the facility administration, medical information office, etc. other than the Radiation Therapy Department in making the connection?
2. If you answered "Yes" to the above question, please provide the name of the specific department. If you know the job title of the person in charge (e.g., person in charge of system information, person in charge of safety management of medical information systems, medical information technologist, etc.), please indicate it as well.
3. Who are the occupations that use Remote Radiotherapy Planning as an individual? (multiple selections allowed)
4. Uses of Remote Radiotherapy Planning (Multiple selections allowed)
5. Who is responsible for the cost of building the system?
6. Who is responsible for network connection costs?
7. Which of the following treatment planning devices implements Remote Radiotherapy Planning?
8. Does your hospital have security guidelines for Remote Radiotherapy Planning?
9. How do you share patient information for treatment planning?
10. Does your hospital maintain records (users, hours of use, etc.) for telework use?
11. Are there any tools used at your hospital for communication when teleworking? (multiple selections allowed)
12. Is there any prior explanation to patients regarding the implementation of remote radiotherapy via telework at your hospital?
13. Which process do you implement in your remote treatment plan? (Multiple selections allowed)
14. Where will the remote treatment plan be implemented? (Multiple selections allowed)
15. For those who responded that the project will be conducted outside of the hospital, either at home or outside of the home, how will the performance fee be handled?

**Questions for both facilities that provide remote radiotherapy and facilities that do not provide remote radiotherapy (Some questions are only for those in Prefectural Base Hospitals for Cancer Treatment)**

1. Do you think Remote Radiotherapy Planning can be a useful tool to increase the number of high precision radiotherapy patients?
2. Are you considering installing a Remote Radiotherapy Planning Support System in the future?
3. Facilities that responded to the above question (plan to introduce, have no plan but would like to introduce), please state the purpose of introduction.
4. What do you think are the main obstacles to the widespread use of Remote Radiotherapy Planning? (Multiple selections allowed)
5. What is your hospital's medical information department's attitude toward the use of Remote Radiotherapy Planning? (If possible, please provide the opinion of the person in charge of the medical information department)
6. Do you think it is possible to relax the facility criteria for IMRT if there is sufficient support for the use of Remote Radiotherapy Planning in addition to one full time physician?(For Prefectural Base Hospitals for Cancer Treatment) If the requirements for remote radiotherapy ((1) collaboration among related facilities and (2) use of telework at facilities) were made mandatory in the requirements for designation as a Prefectural Base
7. Hospital for Cancer Treatment, would it be possible to respond?
8. Facilities that responded to the above question as (not able to respond at this time and will find it difficult to respond in the future), please state the reason(s).
9. If there were an outsourcing organization in the work related to radiation treatment facility, would you be willing to use it as your institution?(Those who answered "Yes" or "Somewhat agree")
10. What kind of work would you like to outsource regarding radiotherapy? (Multiple selections allowed)

Other: Please feel free to provide any comments you may have regarding Remote Radiotherapy Planning.
